# Improving ESP Writing Class Learning Outcomes Among Medical University Undergraduates: How Do Emotions Impact?

**DOI:** 10.3389/fpsyg.2022.909590

**Published:** 2022-06-20

**Authors:** Nan Hu, Min Chen

**Affiliations:** ^1^Foreign Language School, Guangxi Medical University, Nanning, China; ^2^School of Business, Wenzhou University, Wenzhou, China

**Keywords:** ESP writing, emotional effects, learning outcomes, Positive Psychology, medical research articles (MRAs), university undergraduates, *BioCauses corpus*

## Abstract

As English plays a significant role in most professions, improving the English for Specific Purpose (ESP) writing competence allows individuals to participate in the global professional community, which makes ESP writing important for research. However, research on ESP writing is reported to be insufficient, and how factors such as emotions affect ESP writing is rarely and marginally studied. Therefore, this study aimed at investigating how induced emotions influence the learning outcome in ESP writing classes with an emphasis on a particular rhetorical choice among medical university undergraduates. A total of 63 medical university undergraduates were recruited. After the emotional inducement, they were taught with materials selected and adopted from the *BioCauses* corpus and with an explicitly inductive teaching approach. Results revealed that positive emotions positively correlated with better learning outcomes, while negative emotions hindered participants in their learning. The results shed light on the impact of emotional states on ESP/medical research article (MRA) writing, learning, and teaching. Further study implications were provided accordingly.

## Introduction

English “plays a significant role in most professions but perhaps nowhere more so than in medicine, where effective communication is widely recognized as important to clinical outcomes” (Paltridge and Starfield, 2013, p. 243). English communication is also important, for example, for revealing the causes of diseases as well as healing or to exchange novel research findings. Publishing medical research articles (MRAs) in English, therefore, has become the most essential and prestigious tool for professionals to communicate within the medical academic community and to convey novel research findings to a wider world (Swales, 1990; Reimerink, 2006). Also, it is well-recognized that teaching English for Specific Purpose (ESP) writing should not merely control linguistic errors, but also make it become a suitable genre to respond to contexts and practices (Paltridge and Starfield, 2013). Thus, teaching ESP for MRA writing should focus on guiding students toward writing competence regarding particular targets and writing styles. Among many factors that influence the quality of MRAs, causality relations are vital regarding the characteristics and goals of MRAs, namely, discussing and explaining mechanisms, rationales, and principles behind medical research, pharmacological effects, disease healing, and prevention (The Lancet, 2013; Hudzik, 2016). Cao et al. (2018) proposed that cause-effect statements cover about 65% of medical-related articles on average. In addition, distinctive differences in linguistic choices can be found in English academic writing for particular purposes and readers (Paltridge and Starfield, 2013). Thus, guiding medical university students to express causality relations by using MRAs' particular rhetorical choice is indispensable for helping them improve their MRA writing competence to produce adequate articles that respond to the medical contexts.

Pekrun and Linnenbrink-Garcia (2012) stated that “emotions are ubiquitous in academic settings” (p. 259), and it is well-recognized that emotion has a profound effect on language learning (Pekrun et al., 2002; Linnenbrink, 2006; MacIntyre and Gregersen, 2012; Pishghadam et al., 2016). The Affective Filter Hypothesis proposed by Dulay and Burt (1977) also indicates that various affective variables are highly related to foreign language acquisition by either facilitating or hindering the success of acquiring a second language. Besides, numerous studies show that emotions play an influential role in specific areas of foreign language learning, such as semantics, syntax, and language comprehension (Vissers et al., 2013; Liu et al., 2018). However, the influence of emotions on the learning outcome in ESP writing classes, which are intended to improve students' competence especially in MRA writing by understanding and learning the usage of specific linguistic signals to achieve a specific purpose, has remained marginally researched.

Thus, the purpose of this study was to fill this gap by investigating and answering the research question: How do emotions influence the learning outcomes in ESP writing classes with an emphasis on specific linguistic signals functioning as the causality relation markers in MRAs among medical university students? Also, this study aims to provide implications for teaching and future research in accordance with the research findings.

## Literature Review

### The Importance of Causal Relations in MRAs

MRAs are formal, rigorous, and scientific texts that require logical structure, coherence, and structural integrity. Cause-effect relations play an essential role in cohering and representing the core of MRAs (Cao et al., 2018). Furthermore, causality relations function as essential and fundamental relations, because they explain the functioning and mechanisms explored by researchers after observing phenomena, conducting experiments, suggesting treatments, or giving diagnoses. Furthermore, causality relations allow research peers, professionals, and other related personnel to distill essential information and novel knowledge from those articles (Mihăilă and Ananiadou, 2014). This is very helpful for readers to increase reading speed, recall contents, and identify events with higher importance, which significantly reduces the workload of research peers in cohering and understanding the core information of MRAs (Cao et al., 2018). Thus, causality relations play an essential role in constructing MRAs to convey their core information.

#### *Verbs*+*That-Clauses* as the Markers of Causality Relations in MRAs

Causality relations can be conveyed in many ways, and “causal markers are both highly ambiguous and highly variable” in MRAs (Mihăilă and Ananiadou, 2014, p. 24). The tokens with causal meanings such as conjunctives (e.g., because, since), adverbs (e.g., therefore, hence), and prepositions (e.g., because of, due to) can be adopted to mark causality relations (Mo, 2015) [see (1)].

*(1) This acid pH-promoted increase appears to be specific to a subset of PhoP-activated genes (our unpublished results) that includes pmrD* because *expression of the PhoP-regulated slyA gene and the PhoP-independent corA gene was not affected by the pH of the medium*.

(Perez and Groisman, 2007).

Tokens which have no causal meaning can also be employed to convey causality relations. For instance, in sentence (2), the causality relation that SPI-2 genes can be activated because “SsrB binds within SPI-2” is triggered by the word “and,” which possesses non-causal meaning.

*(2) SsrB binds within SPI-2* and *activates SPI-2 genes for transcription*.

(Mihăilă and Ananiadou, 2014, p. 2)

Apart from those two categories of words that belong to the group of close-class words, syntactic markers with words that belong to the group of open-class words, such as *verbs*+*that-clauses*, are “more commonly used as causal [markers]” (Mihăilă and Ananiadou, 2014, p. 2). This is evidenced by the results provided by Mihăilă and Ananiadou (2014) obtained by analyzing the frequency of syntactic markers and *verbs*+*that-clauses* in the *BioCause* corpus. As causality is at the heart of medical knowledge, the *BioCause* corpus annotated with approximately 850 causality relations from open-access, full-text, medical-related journal articles published in the years 2012 to 2018. According to their findings, such syntactic causality markers occupied more than 70% of the total causality relations annotated.

In sentences (3) and (4), “suggesting that” and “suggested that” indicate the causality relations as in the examples. Although the syntactic markers “suggesting that” and “suggested that” do not bear causal meaning (Mihăilă and Ananiadou, 2014), the causality relations are conveyed, and sentences (3) and (4) can be rephrased as (3') and (4'), respectively.

*(3)…location of binding sites varies*, suggesting that *SsrB regulation is unusual*.

*(3') rephrased: …*since *location of binding sites varies, SsrB regulation is unusual*.

(Yoon et al., 2009)

*(4) …DNA disrupted the integrity of the cell envelope causing cell lysis* suggested that *DNA was acting as a cation chelator*.

*(4') rephrased: …*because *DNA disrupted the integrity of the cell envelope which caused cell lysis, DNA could act as a cation chelator*.

(Mulcahy et al., 2008)

**Figure 1:**
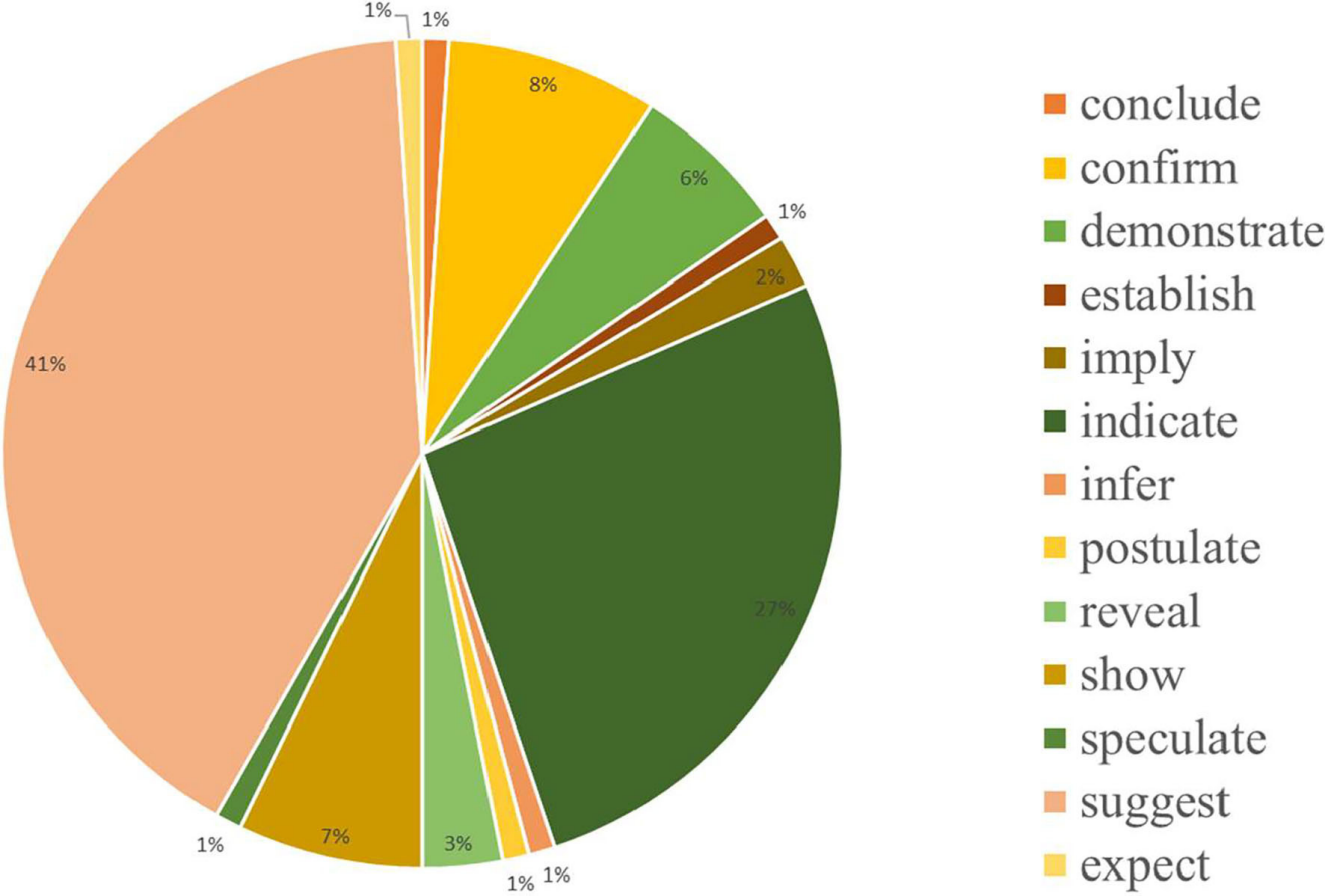
Frequency of verbs used.

It can be discovered based on the above-mentioned examples that *verbs*+*that-clauses* can function either as verbs or -ing adjuncts in sentences when conveying causality relations (Mihăilă and Ananiadou, 2014). Such relations are commonly and largely used when explaining hidden principles or mechanisms in results and discussion sections of MRAs. Therefore, in this study, sentences that employ *verbs*+*that-clauses* to convey causality relations in all result and discussion sections annotated in the *BioCause* corpus are analyzed. Words used and their frequency are shown in Figure 1.

It can be found from Figure 1 that the verbs “suggest” and “indicate” were most commonly used, and other verbs with similar meanings and functions were also employed. Researchers such as Liu and Braine (2005), Mo (2015), and Chen (2019) asserted that in comparison to essays written by English native speakers, Chinese learners who learn English as a foreign language tend to misuse and overuse causal markers. Furthermore, Chinese medical university undergraduates scarcely used *verbs*+*that-clauses* to convey causality relations. Hence, it is vital and indispensable to assist medical university students who desire to join the professional community by publishing sound research articles in English in international medical-related journals in being proficient in adopting specific rhetorical choices to express causality relation, such as *verbs*+*that-clauses*, to produce adequate MRAs.

### Research on ESP Writing and the Influence of Emotions on Language Learning

“In recent years, the field of ESP has become increasingly sensitive to the ways in which texts are written and responded to by individuals acting as members of social groups” (Paltridge and Starfield, 2013, p. 96). However, most of the research on ESP writing mainly focuses on business writing. The studies on English writing for medical purpose (EMP) is either being ignored (Su et al., 2022) or mainly about genres, terminology, hedges, and if-conditional structures (Paltridge and Starfield, 2013). Studies about the impact of other factors such as emotions on ESP, especially on EMP writing, are rare. Although the impact of emotions on ESP learning is insufficiently studied, Flowerdew (2008) proposed that negative emotions may become the burden on the improvement of ESP writing competence.

In terms of general English learning, emotions can be very influential and pervasive during language competence development and language learning and processing (Egidi and Nusbaum, 2012; Driscoll and Powell, 2016; Tyng et al., 2017; Lindquist, 2021). Dewaele and Alfawzan (2018) explained that language learning is closely associated with students' motivation, engagement, memory, and cognitive progress, which are proved to be influenced by emotions. It is well-recognized and well-established that foreign language anxiety plays a negative role in foreign language learners' progress and performance (Liu and Jackson, 2008; MacIntyre and Gregersen, 2012; MacIntyre and Mercer, 2014; MacIntyre and Vincze, 2017; MacIntyre et al., 2019). Dewaele and Alfawzan (2018) and Bensalem (2021) further confirmed such a negative relationship between anxiety and foreign language achievement. Besides, by analyzing a group of 189 foreign language pupils and 152 English learners and users in Saudi Arabia, they discovered that better test results were obtained when students had higher levels of enjoyment in foreign language classes.

Apart from the discussed research, studies concerning subtypes of linguistic knowledge were conducted. Li and Wei (2022) found that enjoyment can be the strongest emotional factor that helped researcher predict English learners' positive achievement by observing 954 learners. However, contrary to the general agreement that positive emotions are associated with better foreign language learning outcomes, the findings of those studies are inconsistent. To be more precise, Pratt and Kelly (2008) examined the effects of emotions on lexical learning and proposed that “people process affective language differently when in positive and negative moods, and lend support to [their position] that emotion and cognition interact during language comprehension” (p. 434). From electroencephalography (EEG) experiments, researchers such as Egidi and Nusbaum (2012), Van Berkum et al. (2013), Egidi and Caramazza (2014), and Lindquist (2021) found that emotions could change real-time language processing, and positive mood seemed to activate referential anticipation that benefited the improvement of reading comprehension, while bad mood abolished such anticipation. Vissers et al. (2010) and Verhees et al. (2015) examined the interaction between syntactic processing and induced emotions (happy vs. sad) by recording event-related potentials and comparing the P600 (the peak in electrical brain activity, which is measured by EEG) effects with subject-verb agreement. The findings revealed that happy emotions were positively related to subject-verb syntactic processing, while bad emotions had a negative influence on the efficacy of processing subject-verb agreement. Vissers et al. (2013) proved that individuals with induced happy emotions performed better on semantic comprehension than the participants with sad emotions. However, Jimenez-Ortega et al. (2012) argued that the happy mood failed to outweigh the other in both semantic and syntactic experiments, which means that higher error rates were found when participants had positive conditions rather than negative emotional conditions. Liu et al. (2018) employed pictures and music that could induce positive emotions (happy) and negative emotions (sad) before teaching Japanese morphosyntax to undergraduates who had not learnt this linguistic aspect previously. The results revealed that when learning Japanese morphosyntax, the induced negative emotions (sadness) increased the accuracy and efficiency of Japanese syntax learning. However, they asserted that such results could not be generalized.

Although there are incompatible research findings and a lack of research on relations between emotions and ESP learning, especially EMP writing competence and, as the research discussed above shows, it is unclear whether those findings, especially the effects of emotions on grasping specific linguistic signals and forms, can be generalized. Numerous studies support the position that positive emotions promote positive language learning outcomes. Hence, it was reasonable to predict that emotional conditions also influence the understanding and learning of *verbs*+*that-clauses* as the specific linguistic markers of causality relations in MRAs, and positive emotions would facilitate such learning. If so, it was reasonable to hypothesize that better teaching outcomes could be obtained through employing emotion manipulation as the strategy in actual teaching of MRA writing, suggesting that the evaluation and examination of emotional effects on such learning are demanding.

Therefore, to fill the gap, this study conducts research on the effects of induced emotions on the learning of one of the most-commonly used MRAs' causality relation markers (i.e., *verbs-that-clauses*), referring to their research design.

## Methods

### Participants

In this research, 63 Chinese native medical university undergraduates in the third academic year were recruited. The research advertisement was released by English teachers of the university, and the participants aged 19–21 years (mean = 19.44; SD = 0.69) took part. After getting the consent forms from all participants, they were randomly divided into two induced-emotion groups: 32 for positive (Group A) and 31 for negative emotions (Group B). Gender distribution (total female: 51.6%; total male: 46.9%) was quite similar across the groups: 17 women and 15 men in Group A, and 16 women and 15 men in Group B. According to the English Curriculum Requirements for non-English major undergraduate students (The Ministry of Education, China, 2020), English is a required subject for all non-English major undergraduate students, and the students took the 45-min English class four times a week. Their English proficiency was evaluated with their scores on the final English exam of the previous semester. Results showed that there was no statistically significant difference (*p* = 0.781). They all passed College English Test (Band 4), which demonstrated that their general English competence was medium or advanced. An extra translation exam (Appendix 1) was employed to test whether the participants are aware of *verbs*+*that-clauses* as causality markers. The results showed that no significant difference was found (*p* = 0.41) among the participants, and they achieved very low scores, indicating that they did not have full understanding and awareness of adopting the target structure to convey causality relations in MRAs.

### Research Design, Materials, and Procedure

#### Research Design

**Figure 2:**
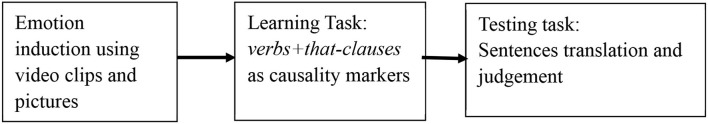
Procedure of the study.

Referring to the study conducted by Liu et al. (2018) and Li and Wei (2022) and considering the purpose of this study, i.e., to explore the effect of emotions on the learning outcomes with an emphasis of a specific rhetorical linguistic signal of casual relations (*verbs*+*that-clauses*) in MRAs, this study includes the following steps (Figure 2): 1. Emotional inducement: induced positive emotion (happy) and negative emotion (sad); 2. Learning task: imparted target rhetorical structures and their purpose; and 3. Testing task: tested learning outcomes.

The learning task was designed with the aim that the participants were trained to understand and learn the usage of target clauses/structures to convey causality relations in MRAs, while the testing task was devised to measure the learning outcomes. Both types of tasks were administrated by the same English teacher *via* the EMP writing training section (~100 min, time for emotion inducement included). Participants' learning outcomes were measured immediately after the treatment *via* “translation” and “judgement” tests (refer to Appendix 2, examples). The reliability and the validity of the final tests were tested in advanced and were 0.87 and 0.78, respectively.

#### Research Materials

**Figure 3:**
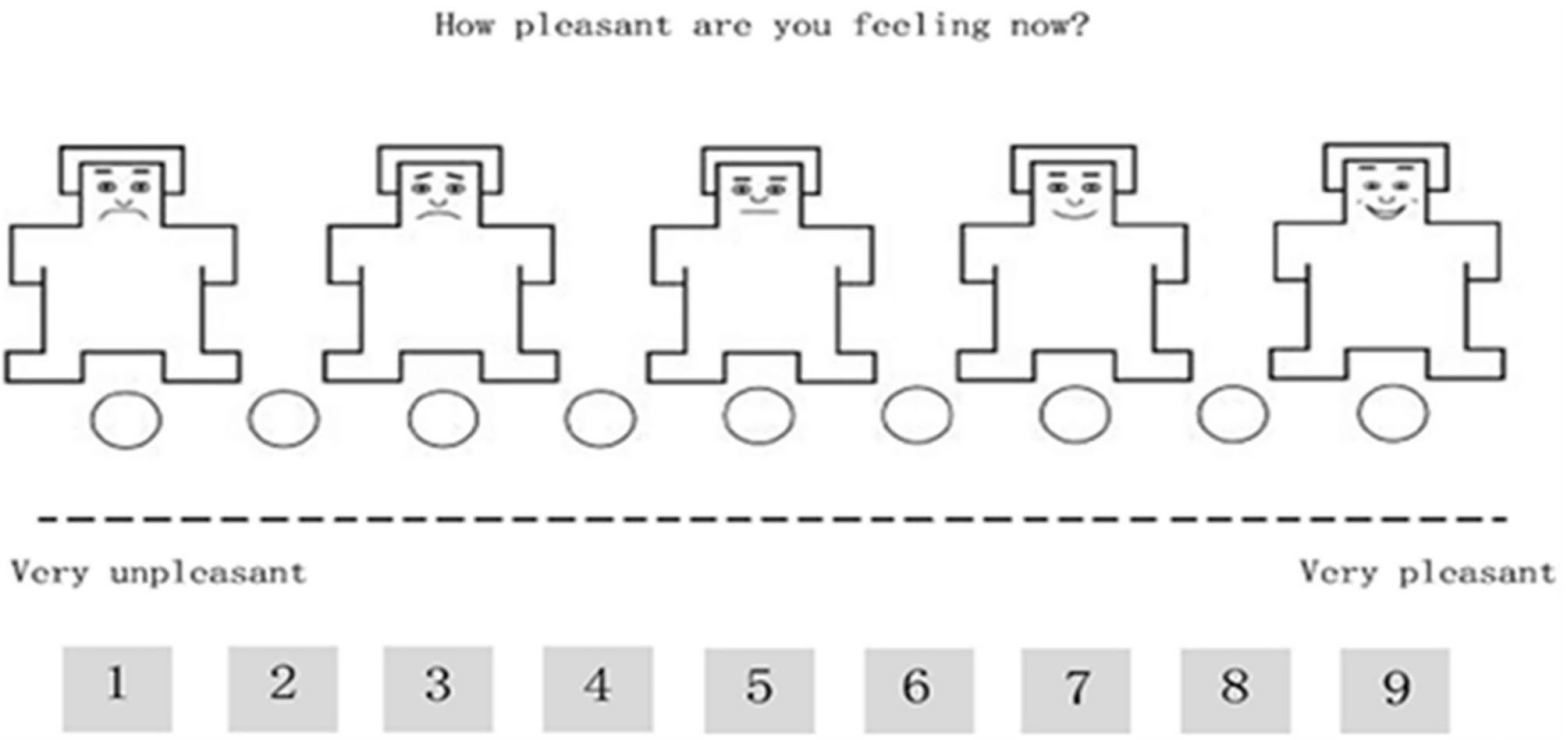
SAM scale on the 9-point scale to evaluate the emotional valence.

Two funny and two sad Chinese video clips between 3 and 5 min in length, as well as 20 funny and 20 sad pictures were employed to induce participants' cheerful (positive) and sad (negative) emotions, respectively. For instance, for inducing the cheerful emotions, the video clip that shows cute dogs was shocked by their owners. Then, the Self-Assessment Manikin (SAM) pictorial rating scale, which also measures arousal and dominance (Bradley and Lang, 1994), was employed to measure participants' emotions, and it has been proved to possess high reliability and validity regarding measuring emotional responses in various situations, including reactions to pictures (Greenwald et al., 1989; Lang et al., 1993; Bradley and Lang, 1994; Geethanjali et al., 2017) and videos (Bradley and Lang, 1994). Due to the aims of this study, only the valence SAM with the 9-point scale (Figure 3) was used.

**Figure 4:**
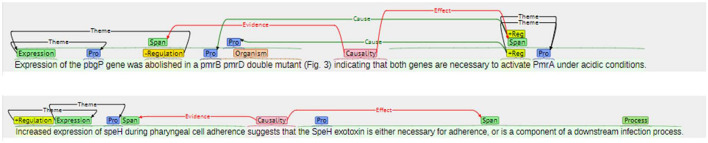
*BioCause* annotation principle.

As writers employ distinctive rhetorical differences for different purposes when writing academic texts in English, authentic and specific articles or writing examples should be utilized to gain an understanding of how professionals communicate and present their research findings effectively and strategically (Liu and Braine, 2005; Lei, 2012; Paltridge and Starfield, 2013). In addition, ESP/EMP articles are always a response to particular readers in particular academic settings, which requires its linguistic choices and forms to serve a specific purpose (Paltridge and Starfield, 2013). The published MRAs can therefore become sound teaching materials, containing commonly used and particular linguistic items as well as rhetorical choices. For this reason, the teaching materials used for this study were obtained from the *BioCause* corpus. The annotation principle employed by the experts who generated *BioCause* is shown in Figure 4.

#### Research Procedure

##### Emotion Induction and Measurement

The pre-test of the target structure was given to make sure that all students had the same knowledge of the target structure to ensure that they would not be misled. Emotion induction was conducted once, and participants' emotional conditions were measured three times *via* the SAM. Higher scores represented higher emotional valence. Participants' emotional conditions were measured before (T0) and after showing the video clips and pictures (T1) to participants in two groups to examine whether the inducement worked effectively. It was measured again before taking the test (T2) to test whether the learning activities influenced their emotional status.

##### The Learning Task

The participants were divided into two groups and the same teaching approaches were adopted by the same teacher in both the groups with the aim that other influential variables can be eliminated to a certain degree. More precisely, the explicitly inductive approach (DeKeyser, 2008) to target knowledge teaching was employed. Eleven sentences with *verbs*+*that-clauses* that convey causality relations were obtained from BioCause and were used as the teaching materials. During the first teaching phase, participants were required to observe some non-medical sentences with simple words, using *verbs*+*that-clauses* as well as other causal markers (e.g., conjunctives, adverbs, and prepositions) to convey causality relations. The differences and similarities were compared in a discussion. The participants were then required to observe six sentences that were obtained from the *BioCause* corpus and their reworded counterparts with conjunctives, adverbs, and prepositions (e.g., since, therefore, because) on hand-outs. The teacher guided participants in analyzing sentences to tell differences and similarities between the original and reworded sentences by drawing Venn diagrams. Also, they received assistance in understanding the meaning of the sentences (medical terms were translated into Chinese), listing the causes and effects described in each sentence. Later, the structure rules and their functions were taught explicitly by the teacher. In the second phase, participants were provided with the rephrased version (the original *verbs*+*that-clauses* were replaced by conjunctives, including “since,” “because,” and “as”) of the remaining five sentences. Then, they were instructed to rewrite those sentences using *verbs*+*that-clauses*, which had been learnt during the first phase. Later, they were offered the original sentences and were required to work in groups to discuss whether they got the correct answers (verbs can be used interchangeably).

##### The Testing Task

In the phase of testing, sentence translation and Yes-No (YN) judgment tasks were devised to test learning outcomes right after the teaching finished. In the YN task, participants were required to judge whether *verbs*+*that-clauses* in the 10 given sentences functioned as causality markers following the judgment task step proposed by Schütze and Sprouse (2014). Such tests are aimed at measuring whether participants establish the awareness of using *verbs*+*that-clauses* to mark causality relations in MRAs. In the translation task, 10 sentences had to be translated from Chinese to English using the taught *verbs*+*that-clauses* (verbs could be used interchangeably). This task was designed to mainly test whether the participants grasped the grammatical rules and functions of *verbs*+*that-clauses*. In doing so, teacher could examine students using the phrases to express casual relations and whether they can produce the correct patterns. The full mark of the test was 20, and three English teachers, who have been teaching in the medical university for more than 8 years, were invited to cross-rate the tests.

## Results

Data were analyzed by employing IBM SPSS Statistics 24, and the significance level was set at 0.01.

### Emotional Inducement

Table 1 summarizes the rating of the initial emotional conditions of the two groups. No significant difference was detected between the two groups before emotion induction, which means that all participants had a similar emotion status (*p* = 0.255).

**Table 1:** Initial emotional conditions of two groups.

**T**_**0**_					
	**Sum of squares**	**df**	**Mean square**	***F***	**Sig.**
Between groups	0.884	1	0.884	1.322	0.255
Within groups	40.767	61	0.668		
Total	41.651	62			

**Figure 5:**
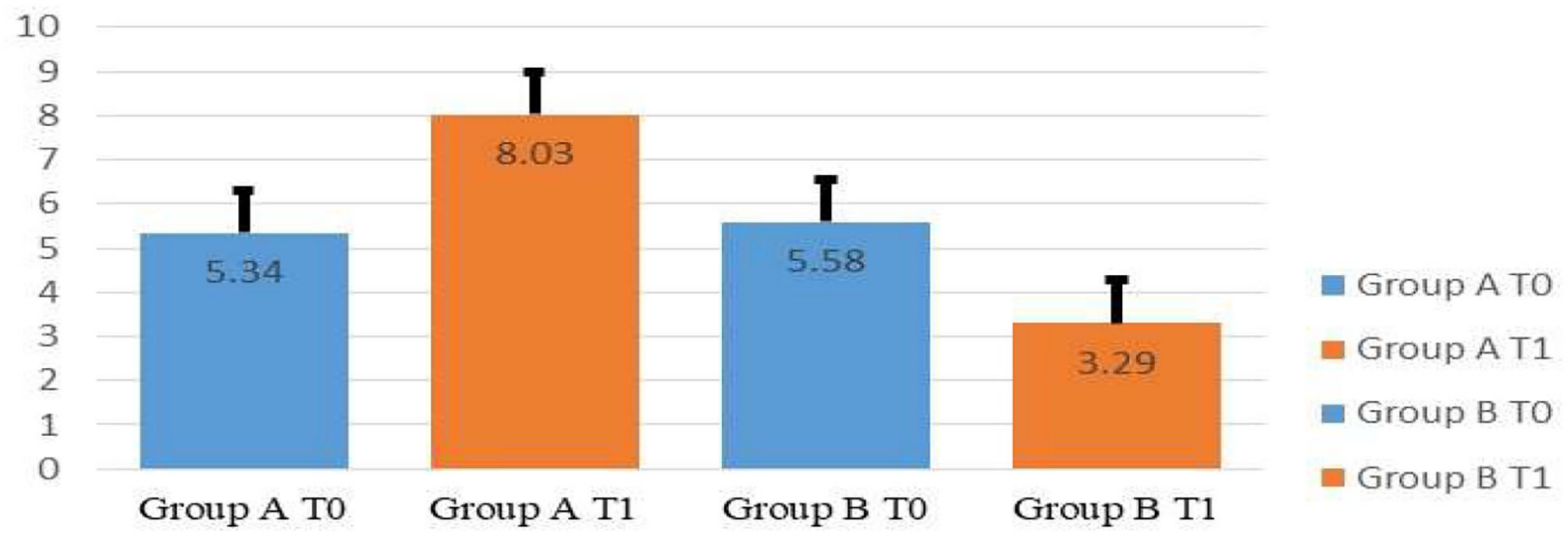
Scores on valence ratings by emotion at T_0_ and T_1_.

After the treatments, the results of the *t*-test analysis revealed that the emotional valence of participants of Groups A and B differed significantly, which means that the approach of inducing emotion was effective (*t*-test results: refer to Figure 5 and Tables 2A,B). Furthermore, the analysis of variance (ANOVA) (Table 3) showed that, after the emotion induction, a significant difference could be observed between the two groups (*p* = 0.00), which further indicates that the emotion induction achieved the anticipated goal. Further correlation analysis was conducted to examine whether the inducement may have difference effect between female and male students. The data suggested that female students' and male students' emotional valence had no significant difference (*p* > 0.05) after conducting emotional inducement (Table 3B).

**Table 2A:** Group descriptive statistics at T_0_ and T_1_.

	**Group**	***N***	**Mean**	**Std. deviation**	**Std. error mean**
T_0_	Group A	32	5.34	0.787	0.139
	Group B	31	5.58	0.848	0.152
T_1_	Group A	32	8.03	0.740	0.131
	Group B	31	3.29	0.864	0.155

**Table 2B:** Comparison of emotional valence before and after the inducement.

			**Levene's test for equality of variances**		***t*** **-test for Equality of Means**						
			***F***	**Sig.**	***t***	**df**	**Sig. (2-tailed)**	**Mean difference**	**Std. Error difference**	**95% confidence interval of the difference**	
										**Lower**	**Upper**
Emotional Valence	Equal variances assumed	Group A	0.993	0.323	−14.071	62	0.000	−2.688	0.191	−3.069	−2.306
		Group B	0.031	0.860	10.537	60	0.000	2.290	0.217	1.856	2.725
	Equal variances not assumed	Group A			−14.071	61.761	0.000	−2.688	0.191	−3.069	−2.306
		Group B			10.537	59.978	0.000	2.290	0.217	1.856	2.725

**Table 3A:** Comparison of emotional conditions of two groups after emotional induction (ANOVA).

**T1**					
	**Sum of squares**	**df**	**Mean square**	**F**	**Sig.**
Between groups	353.914	1	353.914	548.553	0.000
Within groups	39.356	61	0.645		
Total	393.270	62			

**Table 3B:** Emotional conditions of two gender groups after inducement.

**T**_**0**_					
	**Sum of squares**	**df**	**Mean square**	***F***	**Sig.**
Between groups	0.894	1	0.894	1.645	0.366
Within groups	41.767	61	0.668		
Total	43.651	62			

To explore whether the effect of the emotional induction was sustained, and the teaching method influenced participants' emotions, repeated-measures ANOVA was performed, setting the time (second vs. third) as the within-subject variable and the group (Group A vs. Group B) as the between-subjects variable (Tables 4A,B). The results indicate that emotional conditions did not differ significantly after the *verbs*+*that-clauses* were taught (*p* = 0.023). The participants of Group A still possess higher emotional valence than those of Group B, and significant differences existed (*p* = 0.00). The results prove that the effect of emotion induction was sustained in a stable way throughout the learning period, and there was no significant influence of time or the employed teaching method on the participants' emotional conditions.

**Table 4A:** Tests of within-subjects contrasts.

**Within-subjects factors**						
**Time**	**Dependent variable**					
1	T_1_					
2	T_2_					
**Source**	**Time**	**Type III sum of squares**	**df**	**Mean square**	***F***	**Sig**.
Time	Linear	4.022	1	4.022	5.470	0.023
Time ^*^ Group	Linear	7.450	1	7.450	10.133	0.002
Error(Time)	Linear	44.851	61	0.735		

**Table 4B:** Tests of between-subjects effects.

**Between-subjects factors**					
		**Value label**	***N***		
Group	1	Group A	32		
	2	Group B	31		
**Source**	**Type III Sum of Squares**	**df**	**Mean square**	***F***	**Sig**.
Intercept	3785.786	1	3785.786	4921.014	0.000
Group	570.040	1	570.040	740.976	0.000
Error	46.928	61	0.769		

### The Effect of Emotions on *Verbs+That-Clauses* Causality

Participants' learning outcomes were measured by *t*-test analysis to primarily test whether positive emotions (happy) facilitated the target structure learning and its function in understanding MRAs, i.e., whether participants of Group A achieved better learning outcomes, as assumed. The *t*-test analysis showed that test scores obtained after the treatments by the participants of Group A (mean = 18.38; SD = 1.238) were higher than those of Group B (mean = 15.71; SD = 1.596), and significant differences were observed (*p* = 0.00) (Tables 5A,B).

**Table 5A:** Descriptive statistics after the treatments.

	**Group**	***N***	**Mean**	**Std. deviation**	**Std. error mean**
Test score	Group A	32	18.38	1.238	0.219
	Group B	31	15.71	1.596	0.287

**Table 5B:** Comparison of test scores (Group A vs. Group B) after the treatments.

		**Levene's test for equality of variances**		***t*** **-test for equality of means**					
		***F***	**Sig**.	***t***	**df**	**Sig. (2-tailed)**	**Mean difference**	**Std. error difference**	**95% confidence interval of the difference**	
									**Lower**	**Upper**
Test score	Equal variances assumed	1.063	0.307	7.421	61	0.000	2.665	0.359	1.947	3.383
	Equal variances not assumed			7.392	56.569	0.000	2.665	0.361	1.943	3.387

To investigate the relationship between positive emotion (happy) and negative emotion (sad) and the learning outcomes, the Pearson correlation analysis and regression analysis were conducted. The Pearson correlation analysis evidences the high degree of positive relation between positive emotion conditions and better test results (*r* = 0.707, *p* = 0.000). In other words, higher levels of positive emotions can trigger better learning outcomes (Table 6).

**Table 6:** Correlation between emotional valence and test results.

	**Test score**	
T_1_	Pearson Correlation	0.707^**^
	Sig. (2-tailed)	0.000
	*N*	63
T_2_	Pearson Correlation	0.685^**^
	Sig. (2-tailed)	0.000
	*N*	63

** *Correlation is significant at the 0.01 level (2-tailed)*.

To further demonstrate the impact of emotions on the target English structure and its functions' learning outcomes, a regression analysis was conducted. The result also revealed that more favorable learning outcomes were achieved by participants in positive emotion (happy) conditions (Tables 7A,B).

**Table 7A:** The relations between emotional valence and test scores (coefficients^a^)-T_1_.

**Model**		**Unstandardized coefficients**		**Standardized coefficients**	***t***	**Sig.**
		**B**	**Std. Error**	**Beta**		
1	(Constant)	13.945	0.436		31.959	0.000
	T_1_	0.547	0.070	0.707	7.803	0.000

a *Dependent VARIABLE: test scores*.

**Table 7B:** The relations between emotional valence and test scores (coefficients^a^)-T_2_.

**Model**		**Unstandardized coefficients**		**Standardized coefficients**	***t***	**Sig.**
		**B**	**Std. error**	**Beta**		
1	(Constant)	13.688	0.494		27.704	0.000
	T_2_	0.633	0.086	0.685	7.338	0.000

a *Dependent variable: test score*.

The Pearson correlation analysis was conducted to further explore the relationship between emotion conditions (happy vs. sad) and the outcomes of two testing tasks, translation (r1) as well as judgment (r2), as these two testing types reflect whether participants understand the syntactic structures and grasp the awareness of using such structures in marking causality relations in MRAs. The results (Table 8) illustrate that the correlation between positive emotion and translation (r1 = 0.622) is slightly stronger than the one between higher emotional valence and better judgment results (*r*^2^ = 0.375). However, there is no significant difference between the two correlation coefficients (*p* = 0.0673), which means that although the r1 is slightly higher than r2, the extent of the effect of emotion on completing different types of testing tasks did not differ significantly.

**Table 8:** The correlations between emotional valence and translation and judgment scores.

		**Judgement**	**Translation**
T_1_	Pearson Correlation	0.375^**^	0.622^**^
	Sig. (2-tailed)	0.002	0.000
	N	63	63
T_2_	Pearson Correlation	0.392^**^	0.580^**^
	Sig. (2-tailed)	0.001	0.000
	N	63	63

** *Correlation is significant at the 0.01 level (2-tailed)*.

## Discussion and Implications

This study explores the effect of induced positive emotion (happy) and negative emotion (sad) on the understanding and learning of *verbs*+*that-clauses*, which are commonly used as the specific rhetorical choices to mark causality relations in MRAs (Mihăilă and Ananiadou, 2014). In particular, an explicitly inductive approach was employed, authentic teaching materials were selected from the *BioCause* corpus, and the influence of induced positive (happy) and negative (sad) emotions was investigated by employing translation as well as judgment tasks as testing methods.

Following the research design, participants' positive and negative emotions were first induced with happy and sad Chinese video clips and pictures and measured by employing the 9-point SAM scale. The results demonstrate that the emotional manipulation was successful. After the emotion induction, the teacher assisted participants in understanding and learning the structures with which causality relations were conveyed, using authentic teaching materials selected from the *BioCause* corpus. Correlation and regression analyses revealed the positive correlations between positive emotions (happy) and the tests results. Thus, it is fair to assert that positive emotions can facilitate and be positively associated with the understanding and learning of *verbs*+*that-clauses* as causality markers in MRAs. The results are in line with the positions of many researchers that were mentioned in the literature review as well as the initial prediction that such a learning purpose can be enhanced by positive emotions (happy).

Besides, more explanations support the findings. Positive emotions enhance EFL leaners' abilities to notice knowledge they are learning in classrooms and “become more aware of language input,” while “negative emotions can cause narrowing of focus and limit the potential language input” (Dewaele and Alfawzan, 2018, p. 26). Also, Dewaele and Alfawzan (2018) proffered that positive emotions make “students more resilient and hardy during difficult time” (p. 26). Since the results show that the ultimate emotion conditions of two groups' participants did not differ significantly from the induced ones, their emotion conditions remained happy and sad, respectively, during the learning. Sad participants may not be able to accept knowledge input fully and comprehensively. They might not be optimistic either when encountering difficulties while completing the learning tasks. Thus, this finding can support the position that MRA writing competence in terms of using a particular syntactic structure to mark causality relations can be enhanced when medical undergraduates are in positive emotional conditions. When guiding ESP writing, emotion manipulation strategies are therefore recommended for better learning and teaching outcomes.

Results can also be explained based on the Positive Psychology (PP), one of whose aims is to increase learners' wellbeing and positive mental condition. Many research-supported PP-based teaching activities that are employed to encourage learners to flourish inside thereby improving their linguistic and communicative competences and proficiency. By triggering learners' positive mental conditions and health through letting them feel cheerful, students can enhance the “input” and consolidate and store long-term memories about the “input language knowledge” more easily. This is because learners are beneficial from positive psychology, which allows them to tackle emotional overload, develop resistance, and become more optimistic toward the learning outcomes.

For implications, to successfully manipulate the emotions of students, the cultural background, and social contexts, which are influential for the emotion inducement, need to be considered when selecting materials. In this study, the participants in Group A showed much higher emotional valence than participants of the negative emotion group after watching funny and sad Chinese video clips and pictures that could effectively trigger their emotional empathy. Unlike using pictures or video clips in this study, Liu et al. (2018) employed the music “Brandenburg Concerto” as the positive emotion trigger and “Alexander Nevsky: Russian under the Mongolian Yoke” as the negative emotion trigger in their study. Their musical inducement was reported to be not effective, as Chinese participants were not familiar with the music. Thus, culturally and social contextually appropriate materials are recommended to ensure the inducement triggers the anticipated emotions when teaching in different conditions.

Besides, contrary to the results of this study, Liu et al. (2018) suggested that positive-emotion learners, who learnt Japanese morphosyntax, did not perform better than negative-emotion learners. Contrary to the findings of the present research, their negative learners achieved better learning outcomes. According to their explanation, this is because negative-emotion leaners were more motivated to achieve the expected learning goals and were more realistic about the gap between their abilities and desired learning outcomes. Therefore, more studies are necessary to explore whether emotions' effects vary greatly between languages or language areas (e.g., verbs, sentence structure, and morphosyntax). However, with the similar teaching goals, i.e., improving ESP/EMP learning outcomes regarding the writing strategies by using specific linguistic signals in ESP/EMP writings.

In terms of the teaching method, the explicitly inductive approach was used to target ESP writing knowledge teaching in this study. Positive emotions are reported to be highly linked to cognitive perspectives, which intertwined with understanding and mental processes, including memory, thinking, and problem solving (Ashby and Isen, 1999; Winn, 2013). Moreover, Vissers et al. (2013) purported that “positive [emotions] validate[s] accessible cognition and leads to a more [specific to global] category processing of information” (p. 1,027), and such a cognitive process is associated with inductive learning, which is also supported and enhanced by positive emotions (Liu et al., 2018). Therefore, whether to conduct emotional manipulation, whether it could work effectively, and what types of emotions can stimulate better learning outcomes is highly associated with the employed teaching method. This study proves that higher emotional valence is recommended to be triggered when adopting such an explicative specific-general teaching approach, i.e., explicatively inductive teaching procedure. This way, students are led to inductively learn the structure and its functions first, and they are then provided with formulated rules to the applications in practical language use.

Finally, the correlation analyses were conducted to detect the relationships between emotions and results of two types of testing tasks. Both results revealed a positive relationship, and the correlation between emotion and the translation test results (r1) is slightly stronger than the one between emotion and judgment test results (r2). The initial purpose of the analysis was to examine whether the emotion as well as the teaching methods affect students' linguistic structure learning differently and establish awareness of employing particular rhetorical choices for MRAs writing. Although structural learning seemed to relate more closely to positive emotional conditions, there was no statistically significant difference. This result, however, cannot be considered robust evidence for the position that positive emotional conditions made more contributions to the structural learning, and the effects of emotions on the cognitive process of completing different test tasks do not vary. One possible explanation may be that participants' professional knowledge was not sufficient to connect extra textual inferences when judging whether the *verbs*+*that-clauses* marked cause and effect relations in YN judgement tasks. Looking at Q5 (5) as an example shows that (5') is the original sentence selected from the BioCause corpus, and the causality relation was conveyed by “because”. In the judgment test, some participants marked “x,” meaning the “suggested that” clause did not mark causal relations, which is proved to be incorrect. More studies are required to discover the mechanisms behind such relations, and to what extent emotions affect test takers' performance on different categories of ESP learning tasks.

## Conclusion

In ESP writing training, teachers should not merely focus on imparting linguistic knowledge, but also guide students in learning the usage of particular rhetorical choices to communicate effectively and strategically in corresponding fields. However, research on ESP writing is insufficient, and how factors such as emotions affect ESP writing is rarely and marginally studied. Thus, this study examines the influence of induced emotions (happy vs. sad) on the learning outcomes of ESP writing classes with emphasis on the *verbs*+*that-clauses* functioning as causality relation markers in English MRAs among Chinese medical university undergraduates. To be specific, 63 volunteers participated, and published MRAs obtained from the *BioCause* corpus were employed as the teaching materials. After the emotion inducement and teaching with an explicitly inductive approach, their emotion status and learning outcomes were measured and analyzed using the ANOVA and *t*-test. The results proved the success of emotion inducement. The correlation analyses revealed the positive relationships between positive emotions and better learning outcomes, suggesting positive emotions can enhance and facilitate the understanding and learning of *verbs*+*that-clauses* as the causal relation markers in MRAs. Based on the findings, some implications were provided.

However, some limitations still exist. First, this study only tested the emotions on understanding and learning of *verbs*+*that-clauses* in MRAs to convey causality relations, and the results may not be generalizable for ESP/EMP writing training as a whole. More studies are necessary for discovering the correlation between emotions and the learning of other categories of English knowledge. The test methods adopted in this study only examined the immediate learning outcomes, and long-term effects should also be explored. Second, this study only focused on medical university graduates.

Hopefully, further research could focus on and expand the findings of this study to explore the interplay between emotions and other categories of knowledge in ESP/EMP. Despite the limitations, the results shed light on the impact of emotional states on ESP/MRA writing, learning and teaching, and the implications of this study can be referred among the similar teaching and learning conditions.

## Data Availability Statement

The raw data supporting the conclusions of this article will be made available by the authors, without undue reservation.

## Ethics Statement

The studies involving human participants were reviewed and approved by Guangxi Medical University Ethics Committee. The patients/participants provided their written informed consent to participate in this study. Written informed consent was obtained from the individual(s) for the publication of any potentially identifiable images or data included in this article.

## Author Contributions

All authors listed have made a substantial, direct, and intellectual contribution to the work and approved it for publication.

## Conflict of Interest

The authors declare that the research was conducted in the absence of any commercial or financial relationships that could be construed as a potential conflict of interest.

## Publisher's Note

All claims expressed in this article are solely those of the authors and do not necessarily represent those of their affiliated organizations, or those of the publisher, the editors and the reviewers. Any product that may be evaluated in this article, or claim that may be made by its manufacturer, is not guaranteed or endorsed by the publisher.
