# Supplementary material for: Improving ESP Writing Class Learning Outcomes Among Medical University Undergraduates: How Do Emotions Impact?
Source: Front Psychol. 2022 Jun 20;13:909590. doi: 10.3389/fpsyg.2022.909590 (PMC9251421; doi:10.3389/fpsyg.2022.909590)
Supplement: Supplementary file 1 [file Data_Sheet_1.docx]

**Appendix 1. Pre-test questions to measure whether participant possessed the awareness of using *verbs+that-clauses* as the causality markers in MRAs.**

(Scores were given when they express causality relations in the answers, regardless of other errors, such as the meaning of terms was translated incorrectly.)

Translate the following sentences into Chinese.

1. The anticipated appearance of mice could be observed, suggesting that gene knockout was obtained.

2. PI staining was observed in cells exposed to DNA and EDTA, confirming that this treatment was lethal

3. All the convalescent sera tested had SeMac-specific antibody (Fig. 3B), indicating that SeMac is produced in vivo during infection

4. The CAP resistance phenotype of biofilms grown in limiting magnesium (20 microM) was similar to biofilms grown in DNA, confirming that DNA imposes a magnesium limitation stress

5. retS and ladS were also constitutively expressed, indicating that posttranslational modifications are essential for their activity

**Appendix 2. Testing tasks --- some questions examples**

A. Translation (English terminology were provided)

1. 由于掩埋在二聚体界面处的总表面积为1258A2，所以SrcA可作为溶液中的二聚体存在

2. 在测定中包含SeMac不影响马链球菌的调理吞噬作用，所以SeMac不抑制马PMN的马链球菌的调理吞噬作用

3. Rv2623为提供结核分枝杆菌提供抗应激保护，所以RV2623缺陷突变体可能在体内减弱

4. 在生物膜或浮游生物培养中，lux没有明显的DNA诱导的CAP抗性，所以在细胞外DNA存在的情况下，这些基因对CAP抗性是必要的。

B. Yes-No Judgement -- whether the following *verbs+that-clauses* serve as causality markers?

1. There was no significant difference in the percentages of PMNs associated with wild-type and DeltavicK bacteria at both time points and in both horse and rabbit blood (Fig. 3), *indicating that* the DeltavicK mutant retains the ability of S. equi to resist to phagocytosis by PMNs

2. These extracellular toxins target red blood cells to provide access to iron, but often show activity against immune cells, *suggesting that* they contributed to the bacterial response to the immune system of hosts, including phagocytosis by insect blood cells

3. … suggested that if P. luminescens and Y. enterocolitica that interact with the midgut of diverse hosts use the same adhesion and invasive factors

4. the PrtS (Plu1382) secreted by P. luminescens can induce melanization of the hemolymph, which *suggests that* it can probably circumvent the innate immune response of the insect

5. A strain deficient in the pmrA gene was approximately 100,000-fold more sensitive to polymyxin B than the wild-type strain when grown pH 5.8, which *suggested that* this resistance was PmrA-dependent.
